# Development of Herbal Mouthwash Powder Using a Self-Nanoemulsifying Drug Delivery System Containing Galangal Extract and Lemongrass Oil for Oral Candidiasis Treatment

**DOI:** 10.3390/pharmaceutics17050546

**Published:** 2025-04-23

**Authors:** Premnapa Sisopa, Supaporn Lamlertthon, Ruchadaporn Kaomongkolgit, Pratthana Chomchalao, Waree Tiyaboonchai

**Affiliations:** 1 Department of Pharmaceutical Technology, Faculty of Pharmaceutical Sciences, Naresuan University, Phitsanulok 65000, Thailand; premnapa@psru.ac.th; 2 Department of Health and Cosmetic Product Development, Faculty of Food and Agricultural Technology, Pibulsongkram Rajabhat University, Phitsanulok 65000, Thailand; 3 Department of Microbiology and Parasitology, Faculty of Medical Science, Naresuan University, Phitsanulok 65000, Thailand; supapornl@nu.ac.th; 4 The Center of Excellence in Medical Biotechnology, Naresuan University, Phitsanulok 65000, Thailand; 5 Department of Oral Diagnosis, Faculty of Dentistry, Naresuan University, Phitsanulok 65000, Thailand; ruchadapornk@nu.ac.th; 6 College of Medicine and Public Health, Ubon Ratchathani University, Ubon Ratchathani 34190, Thailand; pratthana.c@ubu.ac.th

**Keywords:** SNEDDS, mouthwash, galanga extract, lemongrass oil, denture stomatitis

## Abstract

**Objective:** This study aimed to develop and characterize the physicochemical properties of a self-emulsion drug delivery system (SNEDDS) incorporating galangal extract (GE) and lemongrass oil (LGO). Then, to develop mouthwash powders containing GE- and LGO-loaded SNEDDS (GL-mouthwash powder) as a promising alternative for preventing and treating denture stomatitis. **Methods:** The solubility of GE in various vehicles was determined. Subsequently, pseudo-ternary phase diagrams of the different ingredients, oil (LGO), surfactant (Tween^®^ 80), and co-surfactant (Propylene glycol) were selected to develop the SNEDDS. Then, SNEDDS containing GE and LGO (GL-SNEDDS) were prepared and characterized. The optimized liquid GL-SNEDDS was transformed into GL-mouthwash powder by absorbing onto mannitol and blending with a sweetener. Subsequently, various evaluations including drug recovery, moisture content, emulsification time, stability, anti-*Candida* activity, and in vitro cytotoxicity were performed. **Results:** The developed SNEDDS formulation improved GE and LGO solubility. The optimized GL-SNEDDS exhibited a small droplet size of 148.2 ± 2.1 nm with a polydispersity index of 0.11 ± 0.03 and a zeta potential of 2.14 ± 0.11 mV. In addition, the GL-mouthwash powder demonstrated a high drug recovery of >80% with a low moisture of <10% and exhibited greater physicochemical stability under accelerated conditions. The developed GL-mouthwash powder rapidly formed a stable nanoemulsion within 2 min after reconstitution. Interestingly, GL-mouthwash powder exhibited strong anti-*Candida* activity with no toxicity to human fibroblast cells, which demonstrated superior biocompatibility relative to existing commercial products. **Conclusions:** These findings suggest that GL-mouthwash powder has potential as an alternative prevention and treatment of oral *Candida* infection.

## 1. Introduction

*Candida albicans* is the major pathogenic microorganism causing denture stomatitis (DS). This pathogen inhabits the oral cavity and forms biofilms that can attach to oral mucosa or acrylic denture surfaces [1]. Current prevention of biofilm formation typically involves mouthwash and denture cleaner. While antifungal drugs are available for DS treatment, such as polyenes like nystatin and amphotericin B, these often come with side effects [2,3]. Chlorhexidine (CHX), an antiseptic frequently found in oral hygiene products, effectively reduces *Candida* growth and mature biofilm [4,5]. However, prolonged use of CHX may cause tongue and enamel staining, changes in taste perception, and oral mucosa desquamation [6]. Based on these limitations, more studies are being conducted on natural extracts as alternatives to chemical compounds [7,8]. To this end, a natural mouthwash that maintains an anti-microbial effect with no toxicity to oral tissue is an ideal oral hygiene product.

Plant extracts and essential oils show promising antimicrobial and anti-inflammatory properties, emerging as noteworthy candidates in the ongoing search for alternative DS treatment [9,10]. Galangal *(Alpinia galanga, Zingiberaceae)* and lemongrass *(Cymbopogon citratus, Poaceae)* have gained considerable interest due to their potent activity against a range of oral pathogens, especially *Candida albicans* species [11,12]. Previous research has shown that lemongrass oil (LGO) possessed anti-*C. albicans* biofilm activity through decreasing adhesion and yeast-hyphal transition of *C. albicans* [12,13]. Meanwhile, galangal extract (GE) was observed to suppress germ tube formation in *C. albicans* [14]. Moreover, these two plants have been widely utilized in the food, cosmetic, and pharmaceutical industries for centuries [15,16] and reported as having low toxicity to human cells [17,18]. In preliminary studies, the synergistic activities of the combination of GE and LGO showed an additive effect on the clinical strain of *C. albicans*. However, their poor water solubility and low stability limit their application in medications or cosmetic products that require a solution-based production technology. Therefore, these compounds’ solubility and stability issues must be addressed before they can be effectively incorporated into oral hygiene products.

A self-nanoemulsifying drug delivery system (SNEDDS) is a uniform, anhydrous mixture of oil, surfactant, co-surfactant, and active compound. Upon aqueous dilution with a mild agitation, it spontaneously forms a nanoemulsion with droplet sizes typically ranging from 10 to 200 nm. The advantages of SNEDDS include increased solubility of hydrophobic drugs and improvement of the efficacy of drugs, as well as no toxicity to normal cells [19,20,21]. Unfortunately, SNEDDS in liquid form suffer from poor stability and limited portability. To overcome these limitations, solid SNEDDS have been proposed as alternative formulations. The solid SNEDDS is simply prepared via the adsorption of liquid SNEDDS onto solid carriers, such as microcrystalline cellulose, and colloidal silica [22,23].

Therefore, this study aimed to develop mouthwash powders containing GE- and LGO-loaded SNEDDS (GL-mouthwash powder) as a promising alternative for preventing and treating denture stomatitis. The optimization of the GL-SNEDDS formulation involved characterizing key variables, such as particle size, polydispersity index, appearance, and loading capacity. Then, to produce the GL-mouthwash powder, GL-SNEDDS was adsorbed onto an adsorbent and transformed into a solid form. Furthermore, the efficacy of the GL-mouthwash powder against *Candida* and its cytotoxicity was evaluated.

## 2. Materials and Methods

### 2.1. Materials

Galangal extract (GE) was derived from dried galangal rhizomes via maceration in 95% ethanol, followed by filtration and concentration with a rotary evaporator (Buchi, Switzerland). Thai-China Flavours and Fragrances Industry Co., Ltd. (Phra Nakorn Si Ayutthaya, Thailand) supplied the lemongrass oil (LGO). Standard citral (CT) and 1′-acetoxychavicol acetate (ACA) were obtained from Sigma-Aldrich (St. Louis, MO, USA). Polyethylene glycol 400 (PEG400), Cremophor^®^ RH40, Tween^®^ 80, and propylene glycol (PG) were sourced from Namsiang Co., Ltd. (Bangkok, Thailand). Labrasol^®^ and Transcutol^®^ CG were supplied by Gattefossé (Saint-Priest, France), while mannitol (food grade) was obtained from Vechakit Chemical Co., Ltd. (Phitsanulok, Thailand). HPLC-grade solvents were sourced from LabScan (Bangkok, Thailand), and other chemicals were of analytical grade.

Himedia^®^ (Mumbai, India) supplied Yeast extract peptone dextrose (YPD), Sabouraud dextrose broth (SDB), and Sabouraud dextrose agar (SDA). RPMI-1640, DMEM-F12, Fetal bovine serum (FBS), and Penicillin/Streptomycin were obtained from Gibco™ (New York, NY, USA). MTT reagent was ordered from Amresco Inc. (Solon, OH, USA). Normal human dermal fibroblast, NHDF, cells were supplied from ATCC (ATCC#PCS-201-010, Manassas, VA, USA).

### 2.2. Solubility Studies of GE and LGO

The solubility of GE and LGO in various surfactants (Labrasol^®^, Tween^®^ 80, and Cremophor^®^ RH40) and co-surfactants (Transcutol^®^ CG, PG, and PEG400) was evaluated to identify suitable SNEDDS excipients. Each excipient received excess amounts of GE or LGO and was stirred at room temperature for 24 h. After centrifugation at 5000 rpm for 15 min, the supernatants were analyzed using high-pressure liquid chromatography (HPLC, Shimadzu, Japan) with a Vertisep C18 column (4.6 × 250 mm, 5 µm). An isocratic mobile phase consisting of acetonitrile and 0.1% formic acid in water (60:40 *v*/*v*) was employed at a flow rate of 1 mL/min with 20 µL injection volumes. Detection was performed at 218 nm for ACA, identified as GE’s primary component, and at 233 nm for CT, LGO’s main component. This modified HPLC methodology was adapted from previous studies, which reported limits of detection/quantification (LOD/LOQ) of 0.59/1.79 µg/mL for ACA and 0.12/0.36 µg/mL for CT [24,25,26,27].

In this study, the repeatability was studied by assaying two samples at three concentrations during the same day. The precision results revealed %RSD values below 2.0 for all analytical sets, confirming the consistency and reliability of the test method. ACA and CT concentrations were determined from peak areas using calibration curves within the range of 10–300 µg/mL. Excipients with the highest solubility were selected for further study.

### 2.3. Construction of Pseudo-Ternary Phase Diagrams

The microemulsion region was identified by constructing pseudo-ternary phase diagrams using the water dilution method [28]. Based on prior solubility screening, LGO, Tween^®^ 80, and PG were selected as oil, surfactant, and co-surfactant, respectively. SNEDDS formulations were prepared using surfactant/co-surfactant mixture (S_mix_) ratios of 2.3:1, 2.5:1, and 3:1. Subsequently, oil and S_mix_ were mixed in varying ratios (1:9 to 9:1, *w*/*w*) and diluted with distilled water until turbidity was observed.

### 2.4. Preparation and Characterization of SNEDDS Containing GE and LGO (GL-SNEDDS)

According to the result from pseudo-ternary phase diagrams, six formulations with different ratios of LGO:S_mix_ (Tween^®^ 80: PG) at 1:9 (2.3:1), 1:9 (2.5:1), 1:9 (3:1), 2:8 (2.3:1), 2:8 (2.5:1) and 2:8 (3:1) were prepared and coded as F1-F6, respectively. Then, GL-SNEDDS were formulated by simply dissolving different amounts of GE, i.e., 250 and 300 mg, in 1 g of SNEDDS and continuously stirring for 3 h. Subsequently, 1 mL of each GL-SNEDDS formulation was diluted with 10 mL of DI water in a glass beaker with gentle agitation at room temperature. The process of self-emulsification was visually observed for the appearance of the produced microemulsion immediately and after diluted for 24 h. Triplicate measurements of mean droplet size, polydispersity index (PdI), and zeta potential were conducted. Only formulations exhibiting transparent or translucent characteristics were chosen for subsequent studies.

Mean droplet size and PdI were performed using the dynamic light scattering technique with a Zetasizer (Malvern instrument Ltd., Malvern, UK). All formulations were diluted with distilled water at 1:100 (*v*/*v*). The droplet size analysis was carried out for an 80-s duration.

The Zeta potential was determined using the electrophoretic light scattering technique using a Malvern Zetasizer. The formulations were diluted with distilled water at 1:100 (*v*/*v*) before measuring.

Transmission electron microscopy (TEM, JEOL, Tokyo, Japan) operating at 120 kV was used to examine the morphology of the optimized GL-SNEDDS. Sample preparation involved depositing a droplet onto a carbon-coated copper grid, staining with 2% (*w*/*v*) phosphotungstic acid, and allowing it to air-dry before imaging.

### 2.5. Preparation and Characterization of GL-Mouthwash Powder

The optimized GL-SNEDDS formulation (GE-F5) was prepared by adding 200 mg of GE into 1 g of SNEDDS (F5). Subsequently, the GL-SNEDDS was transformed into a solid by adsorbing it onto mannitol (1:7), a highly porous solid carrier, using a mortar and pestle. Finally, sweetener (xylitol) and preservative (sodium benzoate) were added to the solid GL-SNEDDS to produce the GL-mouthwash powder. The final product contained 1.75% (*w*/*w*) of GE and LGO and was then characterized for drug recovery and moisture content.

Additionally, GL-mouthwash powder (2.85 g) was dispersed in 20 mL of distilled water to obtain a clear nanoemulsion before being characterized in terms of emulsification time, pH, morphology, mean droplet size, polydispersity indexes (PdI), and zeta potentials, as described above.

#### 2.5.1. Drug Recovery (%)

HPLC analysis was used to determine ACA and CT content in GL-mouthwash powder. The process involved dissolving 200 mg of GL-mouthwash powder in 10 mL of methanol, followed by 1-min sonication and centrifugation at 10,000 rpm for 10 min. Prior to HPLC injection, the supernatants were filtered through a 0.22 µm membrane filter, and drug content was analyzed as previously described. Drug recovery percentage was calculated using the formula: (%) drug recovery = (Ca/Ct) × 100, where Ca represents the actual drug content and Ct represents the theoretical drug content in these formulations.

#### 2.5.2. Moisture Content

The moisture content of GL-mouthwash powder was determined in triplicate using a moisture analyzer (Sartorius MA40, Otto-Brenner-Straße, Germany) by measuring the weight loss (%) after drying at 150 °C until constant weight was obtained.

#### 2.5.3. Emulsification Time

The self-emulsification time was evaluated to closely mimic the real-life situation. A 2.85 g of GL-mouthwash was added to 20 mL of distilled water with continuous gentle agitation. The time required for the appearance of a transparent or translucent system was visually observed and recorded [29].

#### 2.5.4. pH Value

A Mettler-Toledo pH meter (Greifensee, Switzerland) was used to measure the pH of GL-mouthwash powder. The sample was diluted with distilled water and mixed to achieve homogeneity, then measured in triplicate at 25 °C.

### 2.6. Antifungal Activity

#### 2.6.1. Strains and Culture Conditions

This study utilized the clinical isolate *Candida albicans* R01, sourced from the Faculty of Dentistry, Naresuan University (Phitsanulok, Thailand). The initial culture was propagated on Sabouraud dextrose agar (SDA) at 37 °C for 24 h. For the purpose of antifungal evaluation, a stock suspension of *C. albicans* R01 was prepared in 0.85% NaCl and its turbidity adjusted to match a 0.5 McFarland standard (corresponding to 1 × 10^6^ cells/mL). To achieve the desired inoculum for the killing kinetic analysis, a 1 mL aliquot of the *C. albicans* R01 suspension was combined with 9 mL of RPMI-1640 medium, yielding a final concentration of 1 × 10^5^ cells/mL.

#### 2.6.2. Killing Kinetics

In this experiment, the reconstituted GL-mouthwash with a GE and LGO concentration of 2.5 mg/mL was used to evaluate the fungal killing rate in comparison to two commercial products: nystatin oral suspension (100,000 Units/mL) and 0.12% chlorhexidine (CHX) antiseptic mouthwash. Blank mouthwash powder (without GE and LGO) was used as a negative control. The time killing kinetics study was carried out using a modified method from Okonogi et al. [21]. The experiment involved the combination of 500 µL of the test sample with 500 µL of a *C. albicans* R01 suspension (1 × 10^5^ cells/mL), followed by incubation at 37 °C. At designated time points (0, 0.5, 1, 5, 15, 30, 60, and 120 min), aliquots were removed. To determine viable cell counts, 10 µL of diluted samples were spread onto SDA plates and incubated at 37 °C for 24 h. Colonies of *C. albicans* R01 were subsequently counted, and killing kinetic curves were constructed by graphing Log CFU/mL against time (min).

#### 2.6.3. Inhibitory Effects on Biofilm Formation of *Candida albicans* Cells in Early, Developing, and Mature Biofilm

The effect of the developed GL-mouthwash on anti-biofilm formation was investigated in comparison with nystatin and CHX commercial products. This experiment was assessed using 3 different protocols, on early (Protocol A), developing (Protocol B), and mature (Protocol C) biofilms following the method of Paulone et al. [30], as below.

Protocol A focused on the assessment of early biofilm formation. In this protocol, 24-h-old biofilms were exposed to the sample (100 µL/well) and incubated at 37 °C for 1, 5, 15, and 30 min. Subsequently, the viable *C. albicans* R01 cells were quantified using the XTT assay.

Protocol B focused on the evaluation of developing biofilms. In this protocol, 24-h-old biofilms were exposed to the samples at 37 °C for 1, 5, 15, and 30 min. After exposure, the biofilms were washed with 200 µL of PBS, followed by adding 200 µL/well of RPMI. The plates were incubated for 24 h at 37 °C. Then, the XTT assay was performed.

Protocol C focused on the assessment of mature biofilms. In this protocol, 48-h-old biofilms were exposed to the samples at 37 °C for 1, 5, 15, and 30 min. Subsequently, the XTT assay was performed.

Each experimental group comprised three replicates.

#### 2.6.4. Determination of Minimum Inhibitory Concentrations (MIC) and Minimum Fungicidal Concentration (MFC)

The broth dilution method was employed to determine the MIC. To prepare the GL-mouthwash dispersion, 2.85 g of GL-mouthwash powder was dissolved in 20 mL of water, yielding final concentrations of 2.5 mg/mL for both GE and LGO. Serial two-fold dilutions of the test samples were prepared in RPMI-1640 medium within microtiter plates. A *C. albicans* R01 suspension, standardized to 1 × 10^6^ cells/mL, was then added to each well and thoroughly mixed. Control wells contained the *C. albicans* suspension in medium without any test compound. After 24 h of incubation at 37 °C, *C. albicans* growth was assessed by measuring turbidity at 600 nm using a microplate reader (Bio-Rad Laboratories Inc., Hercules, CA, USA). The MIC value was identified as the minimum concentration exhibiting 90% growth reduction. The extent of growth inhibition was calculated using the equation: % Inhibition = [1 − (Abs _test,24 h_ − Abs_test,0 h_)/Abs_control,24 h_ − Abs_control,0 h_)] × 100, where Abs_test,24 h_ and Abs_test,0 h_ are the absorbance readings of the test well at 24 and 0 h, while Abs_control,24 h_ and Abs_control,0 h_ are the corresponding absorbance readings for the control well at 24 and 0 h.

The MFC was evaluated by incubating turbidity-free wells on SDA plates at 37 °C for 24 h. The lowest concentration preventing visible growth was determined. All experiments were conducted in triplicate.

### 2.7. Cytotoxicity Studies

The safety of GL-mouthwash powder was evaluated and compared with nystatin and CHX commercial products. The in vitro cytotoxicity test was performed on normal human dermal fibroblast (NHDF) cells using MTT assay and crystal violet staining with modified from Chomchalao et al. [31]. GL-mouthwash powder and its blank mouthwash powder were dissolved in 20 mL of distilled water before testing.

#### 2.7.1. MTT Assay

Cell viability was evaluated using the MTT assay. NHDF cells (1 × 10⁴/well) were seeded in 96-well plates and incubated in DMEM-F12 supplemented with 10% FBS and 1% penicillin/streptomycin at 37 °C and 5% CO_2_. After 24 h, cells were treated with GL-mouthwash, blank mouthwash, 0.12% chlorhexidine, or nystatin suspension (100,000 U/mL) for 30 or 60 s. Untreated cells served as controls. Following treatment, MTT solution (0.5 mg/mL) was added and incubated for 2 h. The medium was then replaced with DMSO to solubilize formazan crystals, and absorbance was measured at 570 nm using a microplate reader (Synergy H1 hybrid, BioTek, VT, USA). Cell viability was expressed as a percentage relative to controls.

#### 2.7.2. Crystal Violet Staining

Crystal violet staining was used to confirm the cell density and integrity. The NHDF cells were seeded and treated with the samples under similar conditions to the MTT assay. After 30 and 60 s exposures, the tested samples were removed and rinsed with phosphate buffer saline. Cells were fixed with 4% paraformaldehyde for 1 h, stained with 0.5% crystal violet for 30 min, rinsed, and air-dried. Then, cell morphology was visualized using a light microscope (EVOS^®^ XL Core, Thermo Fisher Scientific, Waltham, MA, USA).

### 2.8. Stability Study

The stability of GL-mouthwash powder was evaluated under accelerated storage conditions (50 ± 5% °C and 75% ± 5% RH). After 30 days of storage [32], the moisture content, emulsification time, particle size, PdI, zeta potential, pH, color, drug content, and antifungal activity were investigated.

### 2.9. Statistical Analysis

All experiments were conducted in triplicate, and results are presented as mean ± SD. Statistical analysis was performed using a *t*-test or one-way ANOVA, with significance set at *p* ≤ 0.05.

## 3. Results and Discussions

### 3.1. Solubility Studies of GE and LGO

The solubility of active compounds in various surfactants and co-surfactants is critical for optimizing SNEDDS formulations with high drug loading. In this study, low-toxicity non-ionic surfactants were employed due to their high HLB values, which promote stable o/w emulsions [29,33]. Thus, the solubility of ACA (from GE) and CT (from LGO) in the individual surfactant were calculated from the linear equation obtained from HPLC analysis of the ACA standard (y = 46,662x + 99,386, r^2^ = 0.9951) and CT standard (y = 97,850x + 101,573, r^2^ = 0.9979). The results are shown in Table 1. LGO was highly soluble in all surfactants (>1000 mg/mL), while the LGO solubility in co-surfactants was as follows: Transcutol^®^ CG > PEG400 > PG. In addition, GE demonstrated high solubility in LGO (807.31 ± 18.91 mg/mL). The solubility of GE in surfactants followed the order: Labrasol^®^ > Tween^®^ 80 > Cremophor^®^ RH40, and in the co-surfactants: was Transcutol^®^ CG > PG > PEG400. However, GE solution in Labrasol and Transcutol CG exhibited some precipitation after 24 h at room temperature. Therefore, Tween^®^ 80 and PG were chosen as the surfactant and co-surfactant, respectively, to develop the SNEDDS containing GE and LGO through pseudo-ternary phase diagram.

### 3.2. Pseudo-Ternary Phase Diagrams

**Figure 1:**
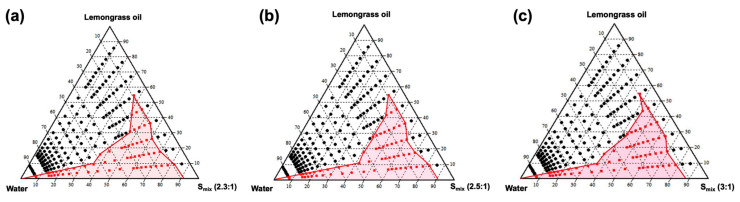
Pseudo-ternary phase diagram of oil (LGO) and S_mix_ (Tween^®^ 80:PG) at different ratios of Tween^®^ 80:PG at 2.3:1 (**a**), 2.5:1 (**b**), and 3:1 (**c**). The colored area represents the SNEDDS region.

Pseudo-ternary phase diagrams of the chosen oil (LGO), surfactant (Tween^®^ 80), and co-surfactant (PG) were constructed using the water dilution method to identify the SNEDDS region. Upon visual observation, SNEDDS was classified as translucent or transparent homogeneous mixtures when diluted with water. The pseudo-ternary phase diagrams revealed that three systems containing surfactant and co-surfactant (S_mix_) ratios of 2.3:1, 2.5:1, and 3:1 exhibited slight differences in the SNEDDS region, as shown in Figure 1a–c. It was observed that the SNEDDS region contracted as LGO concentrations increased and S_mix_ concentrations decreased. Accordingly, all SNEDDS zones were considered, and concentrations of LGO (10% and 20% (*w*/*w*)) and S_mix_ (80% and 90% (*w*/*w*)) were selected for continued investigation.

### 3.3. Physicochemical Characterization of GL-SNEDDS Formulations

Based on the pseudo-ternary phase diagrams, six formulations with different ratios of LGO:S_mix_ (Tween^®^ 80:PG) at 1:9 (2.3:1), 1:9 (2.5:1), 1:9 (3:1), 2:8 (2.3:1), 2:8 (2.5:1) and 2:8 (3:1) were prepared (Table 2). After 100-fold dilution in water, blank SNEDDS formulations formed oil-in-water nanoemulsions, which remained stable with no precipitation after 1 day. The droplet sizes were <100 nm and the PdI values ranged from 0.116 ± 0.042 to 0.274 ± 0.004, indicating a narrow size distribution (Table 3). The zeta potential ranged from −0.8 to −2.4 mV. The results indicated that particle size was controlled by adjusting the oil:S_mix_ and Tween^®^ 80:PG ratios, with size decreasing as surfactant content increased. At a 1:9 oil:S_mix_ ratio, F1–F3 exhibited a mean droplet size of ~11 nm, unaffected by Tween^®^ 80 and PG variations. In contrast, at a 2:8 ratio, F4–F6 showed size variations depending on S_mix_ composition. A higher Tween^®^ 80 concentration reduced droplet size to 96, 76, and 15 nm, respectively, attributed to its ability to lower interfacial energy at the surface of emulsion droplets [33].

To define the GE loading capacity, GE was incorporated into all SNEDDS formulations at 250 and 300 mg/g. All formulations demonstrated effective loading capacity at a GE concentration of 250 mg/g, as shown in Table 3. However, only GE-F2 and GE-F5 demonstrated superior loading capacity at 300 mg/g. After dilution in water, the nanoemulsions formed spontaneously and displayed a yellow-brown color with no visual changes observed after storage for 24 h.

When loaded with 300 mg/g of GE, GE-F2 exhibited a mean droplet size of ~175 nm and GE-F5 exhibited ~148 nm, significantly larger than their blank SNEDDS counterparts (11 nm for F2 and 76 nm for F5). These results confirmed the successful GE entrapment. Zeta potential ranged between 3.4 and −4.3 mV. GE-F2 had the highest PdI value of 0.572 ± 0.003, whereas GE-F5 showed a lower PdI of 0.109 ± 0.028. Since a PdI below 0.3 indicates a monodisperse system with enhanced stability, GE-F5 was selected for further experimentation.

### 3.4. Characterization of GL-Mouthwash Powder

**Figure 2:**
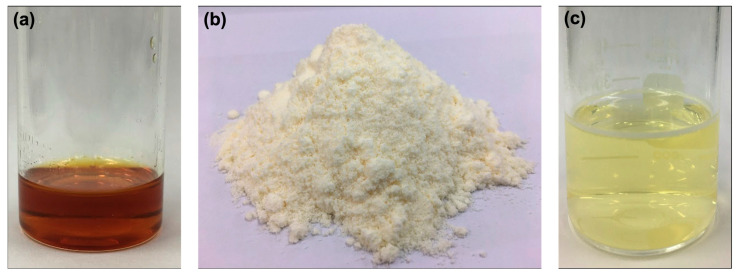
Physical appearance of (**a**) GL-SNEDDS with 200 mg/g of GE, (**b**) GL-mouthwash powder, and (**c**) GL-mouthwash powder after dispersion with water.

Solid SNEDDS was prepared by adsorbing liquid SNEDDS onto the adsorbent. Solid dosage form offers compelling advantages including production simplicity, superior stability, and reproducibility. In addition, recent investigations confirmed that solid SNEDDS significantly enhance the solubility, stability, and oral bioavailability of water-insoluble drugs [22,34]. Due to its superior capacity to load GE and LGO, combined with its small droplet size and narrow size distribution, GE-F5 was selected as the optimal formulation for developing the GL-mouthwash. In our preliminary studies, the antifungal activity of GE and LGO at a concentration of 200 mg/g demonstrated a synergistic effect against *C. albicans* R01. Based on these findings, a 200 mg/g GE formulation was developed for GL-mouthwash by first preparing GL-SNEDDS (Figure 2a), which was then transformed into a solid form using mannitol as an absorbent. The GL-mouthwash powder was subsequently formulated by incorporating a sweetener into the solid form of GL-SNEDDS (Figure 2b). The final product was a light-yellow fine powder with a high percentage of drug recovery for GE (101.32 ± 3.19% *w*/*w*) and LGO (82.54 ± 1.65% *w*/*w*). Nevertheless, the lower percentage recovery of LGO compared to GE might be attributed to LGO evaporation during the preparation process.

The physicochemical characterization of the developed GL-mouthwash powder is shown in Table S1. The GL-mouthwash powder displayed a moisture content of 3.831 ± 0.237%. To obtain a final concentration of GE and LGO at 2.5 mg/mL, 2.85 g of GL-mouthwash was dispersed in 20 mL of water (discussed in Section 3.5). Upon dispersion, nanoemulsion was spontaneously formed (pH 5) with a self-emulsification time of 1.3–1.5 min (Figure 2c). The resulting nanoemulsion possessed a mean droplet size of 50.9 ± 0.7 nm with a PdI value of 0.341 ± 0.013, and zeta potential of −3.663 ± 0.481 mV.

**Figure 3:**
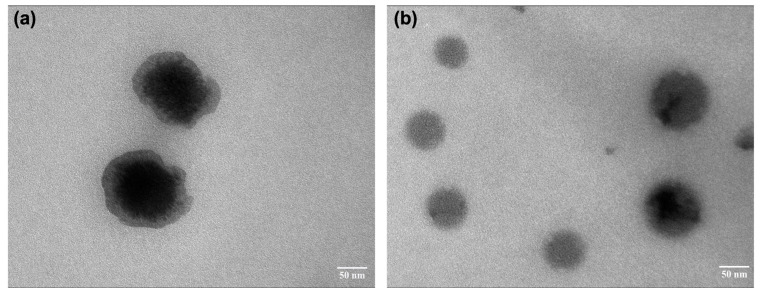
TEM micrographs of GE-F5 of (**a**) GL-SNEDDS; and (**b**) GL-mouthwash powder after being reconstituted in water at a magnification of 50,000.

Furthermore, the TEM micrographs of GL-SNEDDS (GE-F5) and the nanoemulsion obtained from GL-mouthwash are shown in Figure 3a,b. Micrographs revealed that the nanoemulsion from GL-mouthwash had spherical droplets with smooth surfaces and good dispersion similar to those in GL-SNEDDS. This result suggests that GL-mouthwash powder provided nano-size particles with high drug recovery and rapid emulsification in water, thereby making it suitable for preparing the mouthwash solution.

### 3.5. Antifungal Activity

#### 3.5.1. Time Killing Kinetics Study

Time killing kinetic analysis was conducted to determine the rapidity and duration of antifungal activity. In the present study, GL-mouthwash powder was prepared and reconstituted in water to produce a clear mouthwash solution containing 2.5 mg/mL of GE and LGO. According to our preliminary studies, this final concentration corresponds to a 1-fold minimum biofilm inhibition concentration (MBIC) for GE and a 4-fold MBIC for LGO against a clinical isolate *C. albicans* R01. This combination displayed an additive effect, inhibiting biofilm formation by 90%.

**Figure 4:**
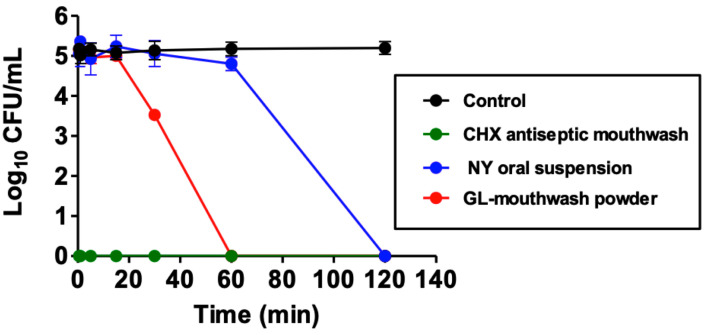
Killing kinetic curves of GL-mouthwash powder, CHX antiseptic mouthwash, and nystatin (NY) oral suspension against *C. albicans* R01 in comparison with the control cell (untreated).

The killing kinetic profiles of each formulation at half concentration against *C. albicans* R01 were compared with the untreated control, as shown in Figure 4. The nanoemulsion derived from GL-mouthwash powder significantly reduced the number of viable *C. albicans* R01 (*p* < 0.05) compared to the control. Among the tested products, CHX antiseptic mouthwash exhibited the fastest fungicidal effect, followed by GL-mouthwash powder and nystatin oral suspension, respectively. GL-mouthwash powder completely eradicated *C. albicans* R01 within 60 min, while nystatin oral suspension required 120 min for complete killing. This result is consistent with previous studies, which reported that mouthwash containing CHX achieved complete killing within 120 s at half the concentration of commercial products [35]. Moreover, Shrestha et al. showed that mouthwash containing CHX eliminated all *C. albicans* strains more rapidly than mouthwash containing thymol (essential oil) [36]. Furthermore, CHX showed superior fungicidal activity over antifungal drugs (amphotericin B and nystatin), requiring less time to eliminate *C. albicans* in time-kill assays [37].

#### 3.5.2. Inhibitory Effects on Biofilm Formation of Candida Cells in Early, Developing, and Mature Biofilm

The persistence of *C. albicans* in the oral cavity is associated with biofilms, which possess higher antifungal resistance compared to planktonic cells. Consequently, investigating the behavior of biofilm-organized cells when exposed to the GL-mouthwash product is important. In particular, the contact time range of 0.5–30 min was designed to mimic real-life practical application times of mouthwash as closely as possible. The effects of GL-mouthwash powder, nystatin oral suspension, and CHX mouthwash on *C. albicans* R01 cells embedded in early, developing, and mature biofilm were assessed by protocols A, B, and C, as described above.

**Figure 5:**
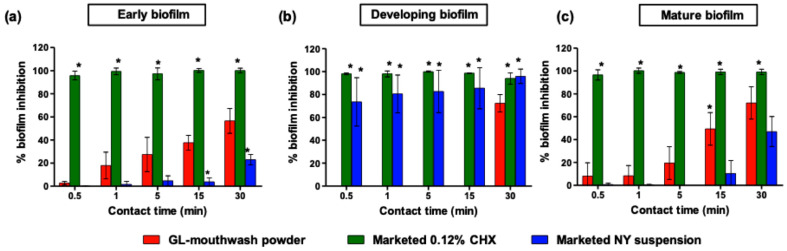
Percentage of biofilm inhibition of each mouthwash at different times on *C. albicans* R01 cells embedded in (**a**) early, (**b**) developing, and (**c**) mature biofilm. Asterisks (*) demonstrate the significant difference between GL-mouthwash powder and CHX antiseptic mouthwash and NY oral suspension (*p* < 0.05).

As shown in Figure 5, CHX mouthwash demonstrated superior antifungal activity against *C. albicans* R01 biofilms at all stages (early, developing, and mature) and across all contact times, followed by GL-mouthwash powder and nystatin oral suspension. The inhibitory effect of GL-mouthwash powder against *C. albicans* R01 in early and mature biofilm increased with longer contact times (Figure 5a,c). However, it effectively inhibited the developing biofilm only at a 30-min contact time (Figure 5b).

CHX, known as the most effective agent against dental biofilm, disrupts fungal cells by binding to negatively charged cell walls, causing cellular leakage and leading to irreversible fungal death [6]. In this study, CHX mouthwash eliminated biofilm formation at all stages by more than 90% within 30 s. These findings align with a previous report showing that 0.2% CHX mouthwash effectively inhibited *Candida* biofilm at a short contact time [24].

NY oral suspension is widely used to treat oral candidiasis. NY, a broad-spectrum antifungal activity, binds to ergosterol in the fungal cell membrane, increasing ion permeability and resulting in cell death. In this study, NY oral suspension showed limited efficacy against early and mature biofilm, depending on the contact duration, but reduced *C. albicans* R01 in developing biofilm by approximately 70% within 30 s. This may be due to its prolonged post-antifungal effects [31].

GL-mouthwash powder, containing GE and LGO-loaded SNEDDS, exhibited moderate efficacy against early and mature biofilm, depending on the contact duration. This antifungal activity can be attributed to the specific mechanisms of ACA and citral as the main active agents in GE and LGO, respectively. They disrupt fungal cells by targeting cell membrane integrity and inhibiting the morphological transition between yeast and hyphal forms (dimorphism) in pathogenic fungi. [32,33]. Moreover, the nanoemulsions derived from the GL-mouthwash significantly enhance therapeutic efficacy through multiple mechanisms: they markedly increase the water solubility of GE and LGO and enhance active ingredients’ penetration into biofilms. However, in the developing stage of biofilm, GL-mouthwash demonstrated significant antifungal activity only at the 30-min contact time. It is possible that the increased plasticity of *Candida* cells during the biofilm developing phase may contribute to their decreased susceptibility to GE and LGO [30].

**Figure 6:**
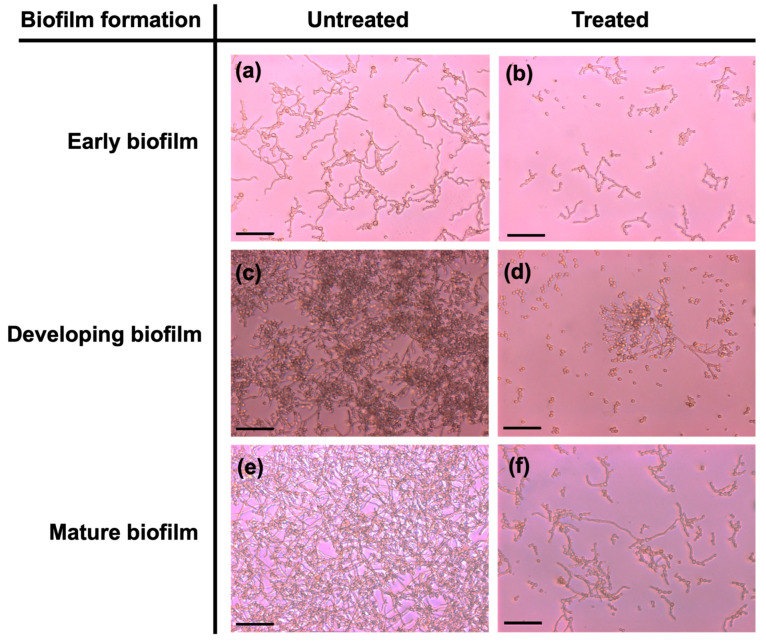
The morphology of *C. albicans* R01 biofilm formation was observed in three stages—early, developing, and mature—both untreated (**a**,**c**,**e**) and treated (**b**,**d**,**f**) with GL-mouthwash powder, using an optical microscope. Data are presented as means ± SD (*n* = 3). One representative image from three independent experiments is shown at 400× magnification, with a scale bar of 50 μm.

To confirm the antibiofilm activity of GL-mouthwash powder, *C. albicans* R01 cells embedded in early, developing, and mature biofilm were treated for 30 min and subsequently examined under optical microscopy (Figure 6). Untreated *C. albicans* R01 biofilm in all three stages demonstrated a densely packed biofilm, as shown in Figure 6a,c,e. On the contrary, GL-mouthwash powder treatment resulted in a marked reduction of viable cell density compared to the untreated cells, as shown in Figure 6b,d,f.

### 3.6. Cytotoxicity Studies

**Figure 7:**
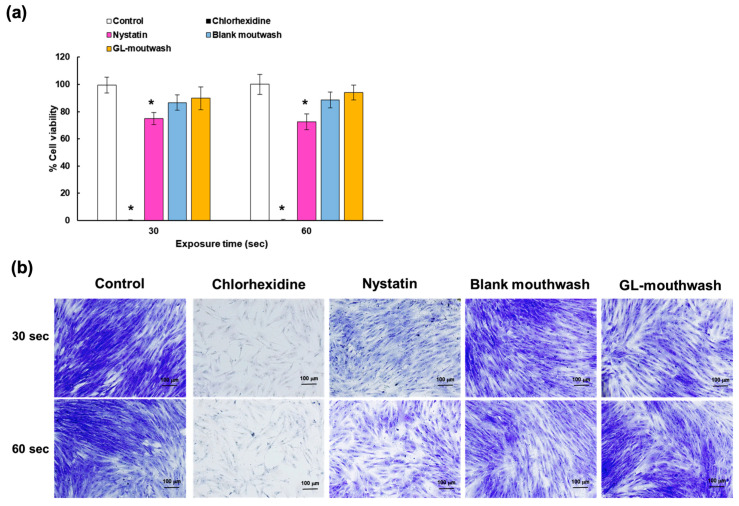
Cytotoxicity of GL-mouthwash investigated in NHDF cells over time; (**a**) cell viability using MTT assay, and (**b**) cell integrity and density using crystal violet staining. Asterisks (*) demonstrate the significant compared with control (*p* < 0.05).

The ideal mouthwash should be safe and have no toxicity. Therefore, the cytotoxicity of GL-mouthwash was investigated in comparison with two commercial mouthwashes including CHX and nystatin. The application of all tested formulations, including the two commercial products (instructed for gargling for 1 min twice daily), was standardized to a 1-min incubation period. This duration was chosen to simulate the typical contact time during real-life mouthwash usage by patients. The cytotoxic effects on the NHDF cells using the MTT assay are presented as the percentage of cell viability in Figure 7a. The results showed that 0.12% CHX caused 100% cell death within 30 s, indicating severe cytotoxicity to fibroblasts. Similarly, Alleyn et al. reported significant cytotoxicity in ligament fibroblasts after 3 min of CHX exposure [38]. James et al. also found that CHX dilution of ≥0.02% exhibited cytotoxic effects on human fibroblast, myoblast, and osteoblast cells [39]. Furthermore, NHDF cells exposed to nystatin suspension for 30 and 60 s reduced cell viability to ~74% with significantly lower than the control cells, suggesting mild cytotoxic. Although nystatin is the drug of choice for the treatment of oral candidiasis, it can bind weakly to cholesterol, potentially causing toxic effects on mammalian cells [40]. Interestingly, GL-mouthwash and its blank exhibited cell viability > 85% after 60-s treatment, indicating no cytotoxic effects on NHDF cells.

In addition, the safety of GL-mouthwash was confirmed by crystal violet staining, as illustrated in Figure 7b. This simple method can be used to observe the density and morphology of viable cells which are stained and attached to a cell culture plate. Dying cells lose adhesion and detach, leading to a reduction in crystal violet staining in culture. The result revealed that the cells exposed to CHX and nystatin had significantly lower stained cells compared to the control, indicating cell death and loss of adherence to the culture plate. In contrast, NHDF cells treated with GL-mouthwash and its blank displayed no significant differences in cell density or morphology compared to the untreated control cells. Therefore, these results indicated that GL-mouthwash demonstrated no toxicity, was safer, and more biocompatible than commercial mouthwashes for oral application.

Integrating GE and LGO into the SNEDDS mouthwash formulation demonstrates a novel approach to integrating natural antimicrobial efficacy with advanced drug delivery technology. Compared to conventional mouthwashes, such as chlorhexidine-based formulations, the SNEDDS offers several potential clinical advantages. Chlorhexidine, with its broad-spectrum antimicrobial activity, is the clinical standard. However, its side effects, including tooth staining, mucosal irritation, and taste alteration, can hinder patient compliance with long-term use. Similarly, conventional antifungal agents like nystatin and miconazole are limited by drug resistance and the need for frequent administration. In contrast, our novel formulation offers a natural, antimicrobial alternative with anti-inflammatory and antioxidant benefits. This could be a significant step in managing fungal infections and mucosal inflammation. Its natural origin may also reduce adverse effects and improve patient acceptance, particularly among those seeking plant-based options. However, further in vivo and clinical investigations are needed to validate its safety, efficacy, and real-world potential, especially in conditions such as oral candidiasis and mucositis. This ongoing research offers a promising opportunity for advancement in the field.

### 3.7. Stability Study

Accelerated storage conditions of 50 °C ± 5 °C and 75% ± 5% RH for 30 days were used to expedite the chemical degradation of the drug substance or physical changes of the product under extreme conditions. These studies help predict long-term stability under normal storage conditions [41]. The physical and chemical stability of GL-mouthwash powder is summarized in Table 4. After 30 days under accelerated storage conditions, no significant physical changes were observed compared to the freshly prepared sample. However, a minor reduction in the moisture content and pH, along with a slight increase in particle size, was noted. Additionally, the chemical stability of ACA and CT, the main active components of GE and LGO, respectively, was also evaluated. After a 30-day storage under accelerated conditions, the remaining active contents were ~90% of ACA and ~ 70% of CT. These findings suggest that elevated temperature conditions could accelerate oxidative degradation of CT, consistent with previous research [42]. In addition, the GL-mouthwash formulation possessed antifungal activity against *C. albicans* R01, with MIC/MFC values of 8-fold serial dilution, equivalent to GE and LGO of 0.3125 mg/mL, consistent with those observed in the initial formulation. Thus, the findings suggested that GL-mouthwash powder could maintain physical and chemical stability under long-term storage conditions. Nevertheless, future studies should be conducted on the stability of GL mouthwash powder at ambient temperature for long-term storage.

## 4. Conclusions

In this study, SNEDDS containing GE and LGO were successfully formulated to enhance the herbal extracts’ solubility. Then, it was further developed into GL-mouthwash powder by absorption with mannitol, followed by blending with a sweetener, to improve the stability and portability. The GL-mouthwash powder formulation possessed a high GE loading capacity, excellent active pharmaceutical ingredient recovery (>80%), low moisture content (<10%), and maintained good physical and chemical stability under stress conditions. After reconstitution in water, this formulation rapidly self-nanoemulsified within 2 min to form a clear solution without precipitation. In addition, the developed GL-mouthwash formulation revealed no toxicity to normal human fibroblast cells and effectively inhibited planktonic fungal cells and biofilm formation of *C. albicans*. Overall results confirmed the potential of GL-mouthwash powder as a promising herbal mouthwash alternative for the prevention and treatment of oral candidiasis.

## Figures and Tables

**Table 1:** Solubility of GE and LGO in various vehicles.

Ingredients	Solubility (mg/mL)	
	GE	LGO
**Oil:**		
Lemongrass oil	807.31 ± 18.91	NT
**Surfactant:**		
Tween^®^ 80	535.24 ± 6.81	1001.53 ± 54.02
Labrasol^®^	657.44 ± 11.39	1047.56 ± 20.20
Chemophor^®^ RH40	476.80 ± 16.61	1031.80 ± 45.97
**Co-surfactant:**		
Propylene glycol	672.04 ± 23.00	239.32 ± 13.61
Transcutol^®^ CG	701.02 ± 33.55	1002.19 ± 38.76
Polyethylene glycol 400	232.53 ± 5.72	725.52 ± 26.98

NT, not tested.

**Table 2:** Composition of selected SNEDDS formulations.

Formulations	Ratio of Oil:S_mix_	LGO:Tween^®^ 80:PG (% *w*/*w*)
F1	1:9	10:63:27
F2	1:9	10:64:26
F3	1:9	10:67:23
F4	2:8	20:56:24
F5	2:8	20:57:23
F6	2:8	20:60:20

**Table 3:** Effect of GE loading amount in SNEDDS formulations on appearance, particle size, and polydispersity index (PdI) of the obtained nanoemulsions after dilution with water.

Formulations	GE Amount (mg/g)	Appearance After 24 h Storage	Particle Size (nm) (Mean ± SD)	Mean PdI (Mean ± SD)	Zeta Potential (mV) (Mean ± SD)
F1	-	Transparent	11.74 ± 0.08	0.17 ± 0.06	−0.79 ± 0.04
F2	-	Transparent	11.62 ± 0.43	0.19 ± 0.06	−2.22 ± 0.21
F3	-	Transparent	11.02 ± 0.13	0.12 ± 0.04	−1.59 ± 0.24
F4	-	Translucent	95.91 ± 0.18	0.14 ± 0.01	−1.43 ±0.11
F5	-	Translucent	75.89 ± 0.70	0.28 ± 0.00	−0.85 ± 0.12
F6	-	Transparent	14.75 ± 0.08	0.28 ± 0.01	−1.99 ± 0.02
GE-F1	300	Turbid	399.10 ± 87.28	0.38 ± 0.02	1.19 ± 0.21
GE-F2	300	Translucent	175.56 ± 1.41	0.57 ± 0.00	0.77 ± 0.08
GE-F3	300	Separate	ND	ND	ND
GE-F4	300	Separate	ND	ND	ND
GE-F5	300	Translucent	148.2 ± 2.1	0.11 ± 0.03	2.14 ± 0.11
GE-F6	300	Separate	ND	ND	ND
GE-F1	250	Transparent	17.68 ± 3.79	0.23 ± 0.06	−1.20 ± 0.31
GE-F2	250	Transparent	30.11 ± 15.59	0.19 ± 0.09	−1.09 ± 0.18
GE-F3	250	Transparent	12.71 ± 0.06	0.16 ± 0.02	−4.13 ± 0.18
GE-F4	250	Translucent	130.70 ± 1.04	0.09 ± 0.02	2.54 ± 0.68
GE-F5	250	Translucent	126.97 ± 1.50	0.10 ± 0.01	1.55 ± 0.24
GE-F6	250	Translucent	142.43 ± 0.57	0.11 ± 0.03	0.84 ±0.18

ND, not determined.

**Table 4:** Characteristics of GL-mouthwash powder before and after a 30-day storage under accelerated conditions.

Characterizations	GL-Mouthwash Powder	
	Initial	Stored at 50 °C
Moisture content (%)	3.94 ± 0.11	2.89 ± 0.37 *
emulsification time (min)	1.3–1.5	1.3–1.5
pH value	5.20 ± 0.01	4.86 ± 0.02 *
Particle size (nm)	50.91 ± 0.67	118.33 ± 2.08 *
polydispersity index	0.34 ± 0.01	0.35 ± 0.01
Zeta potential (mV)	−6.18 ± 0.31	−6.21 ± 0.26
Antifungal activity (MIC/MFC)	8-fold serial dilution	8-fold serial dilution

Asterisks (*) indicate statistically significant differences between freshly prepared GL-mouthwash powder and those stored under accelerated conditions (*p* < 0.05).

## Data Availability

All raw data will be made available upon request to the corresponding author.

## Supplementary Materials

The following supporting information can be downloaded at: https://www.mdpi.com/article/10.3390/pharmaceutics17050546/s1, Table S1: The characterization of GL-mouthwash powder.
