# Supplementary material for: Development of Herbal Mouthwash Powder Using a Self-Nanoemulsifying Drug Delivery System Containing Galangal Extract and Lemongrass Oil for Oral Candidiasis Treatment
Source: Pharmaceutics. 2025 Apr 23;17(5):546. doi: 10.3390/pharmaceutics17050546 (PMC12114958; doi:10.3390/pharmaceutics17050546)
Supplement: Supplementary file 1 [file pharmaceutics-17-00546-s001.zip › pharmaceutics-3567425-supplementary.pdf]

Supplementary data

Development of Herbal Mouthwash Powder using a Self-Nanoemulsifying Drug Delivery System Containing Galangal Extract and Lemongrass Oil for Oral Candidiasis Treatment

Premnapa Sisopa, Supaporn Lamlertthon, Ruchadaporn Kaomongkolgit, Pratthana Chomchalao and Waree Tiyaboonchai*

Table S1 The characterization of GL-mouthwash powder

Characterization	Values
Moisture content (%)	3.83 ± 0.24
emulsification time (min)	1.30 -1.50
pH value	5.20 ± 0.00
Particle size (nm)	50.91 ± 0.67
polydispersity index	0.34 ± 0.01
Zeta potential (mV)	-3.67 ± 0.48
